# GelMA@ginsenoside Rb3 Targets Inflammatory Microenvironment in Periodontitis via MAPK Pathway

**DOI:** 10.3390/gels11080648

**Published:** 2025-08-15

**Authors:** Jinmeng Sun, Minmin Sun, Zekun Li, Luyun Liu, Xinjuan Liu, Yuhui Sun, Gang Ding

**Affiliations:** School of Stomatology, Shandong Second Medical University, Weifang 261053, China; sunjinmeng07@163.com (J.S.); sunminmin@sdsmu.edu.cn (M.S.); zekun_lee@163.com (Z.L.); liuluyun0120@163.com (L.L.); lxj20230834@163.com (X.L.); sunyh03xy@163.com (Y.S.)

**Keywords:** periodontitis, periodontal ligament stem cells, ginsenoside Rb3, GelMA, MAPK pathway

## Abstract

This study aims to develop a gelatin methacryloyl (GelMA)-based ginsenoside Rb3 (G-Rb3) drug delivery system and investigate its application in the treatment of periodontitis and the underlying mechanisms. Periodontal ligament stem cells (PDLSCs) were obtained and identified. The appropriate concentration ranges of G-Rb3 and lipopolysaccharide (LPS) were investigated by the CCK-8 experiments. Quantitative RT-PCR, ELISA, and Western blot were performed to assess the effects of GelMA@G-Rb3 on LPS-treated PDLSCs. The possible mechanisms were determined through network pharmacology analysis and Western blot. The therapeutic effects of GelMA@G-Rb3 in rat periodontitis animal models were systematically evaluated using Micro-CT, H&E staining, Masson staining, and immunofluorescence staining. PDLSCs were successfully isolated and characterized. The in vitro results indicated that GelMA@G-Rb3 significantly alleviated LPS-induced inflammation in PDLSCs by inhibiting the p38/ERK signaling pathway and activating the PI3K/AKT signaling pathway. In vivo experiments confirmed that GelMA@G-Rb3 significantly reduced alveolar bone resorption, and promoted periodontal tissue regeneration, while simultaneously demonstrating significant regulatory effects on the MAPK signaling pathway. This study demonstrated the efficacy of the GelMA@G-Rb3 system in modulating the inflammatory responses of periodontitis and improving the periodontal tissue regeneration, which establish a solid foundation and proposed innovative therapeutic approaches for the treatment of periodontitis.

## 1. Introduction

Periodontitis is a chronic inflammatory disease that affects the gingiva, periodontal ligament, alveolar bone, and cementum. Its primary clinical manifestations include gingival inflammation (redness, swelling, and bleeding), progressive alveolar bone resorption, and eventual tooth loosening or loss, reflecting the characteristic tissue destruction cascade of the disease. As the leading cause of adult tooth loss, periodontitis is significantly associated with major systemic diseases, particularly diabetes and cardiovascular conditions [1]. According to the Global Oral Health Status Report (2022), severe periodontitis affects approximately 19% of the global population aged 15 years and older, representing over 1 billion cases worldwide [2]. Poor oral health imposes substantial social, economic, and environmental burdens, and with the aging global population and rising life expectancies, it is projected that the global impact of severe periodontal disease will continue to grow [3]. Although the exact causes of periodontitis are still unclear, dental biofilm is considered the primary initiating factor. Dental biofilm is a structured microbial community embedded in an extracellular polymeric matrix, which develops through sequential stages: (1) acquired pellicle formation on tooth surfaces, (2) reversible adhesion of pioneer bacteria (mainly streptococci), (3) irreversible adhesion and microcolony formation, (4) biofilm maturation with increasing microbial diversity (including periodontal pathogens like Porphyromonas gingivalis), and (5) dispersion of microorganisms. The imbalance between this complex oral microbiota and host immune response can induce inflammatory reactions and tissue destruction, ultimately determining the progression of periodontitis [4].

The field of Traditional Chinese Medicine has attracted growing interest as a source of novel therapeutics, particularly given that many modern pharmaceuticals originate from herbal medicines and natural products [5,6]. Ginseng, a member of the Araliaceae family and the Panax genus, is a perennial herb that boasts a rich history and substantial medicinal properties [7]. Among the key active constituents of ginseng, gin senoside Rb3 (G-Rb3) has garnered increasing research attention from the researchers for their diverse biological activities and pharmacological impacts. It has been established that ginsenosides exhibit a range of pharmacological benefits, including immunomodulation [8,9], and anti-inflammatory action [10,11], among others [12,13]. Our previous studies demonstrated that G-Rb3 possesses potent anti-inflammatory capabilities, efficiently suppressing the release of inflammatory mediators and the generation of osteoclast, ultimately attenuating alveolar bone resorption in experimental periodontitis rats [14,15].

Gelatin methacryloyl (GelMA) is a biocompatible, photocrosslinkable hydrogel derived from gelatin and methacrylic anhydride (MA) [16,17]. Its primary component, gelatin (>99%), is obtained from hydrolyzed collagen and provides excellent cytocompatibility and biological properties resembling the native extracellular matrix. The methacryloyl groups introduced by MA react with amino and hydroxyl groups on gelatin molecules, enabling photocrosslinking to form stable networks. Additionally, as a local drug delivery system with sustained release capability to prolong drug action duration, GelMA is highly suitable for minimally invasive procedures in clinical practice based on its injectability and photocrosslinking properties, which have been validated in numerous studies [18,19,20,21]. GelMA can be directly delivered to specific treatment sites via simple injection and then rapidly solidified through photocrosslinking technology to achieve immediate treatment.

Herein, we integrated GelMA with G-Rb3 to develop a composite hydrogel that encapsulates G-Rb3, which is injectable and amenable to photocrosslinking. This composite hydrogel was capable of modulating the inflammatory response in periodontal ligament stem cells (PDLSCs) triggered by lipopolysaccharide (LPS). In this study, we demonstrated the significant therapeutic efficacy of the GelMA@G-Rb3 composite hydrogel in a rat model of periodontitis.

## 2. Results and Discussion

### 2.1. Culture and Identification of PDLSCs

**Figure 1:**
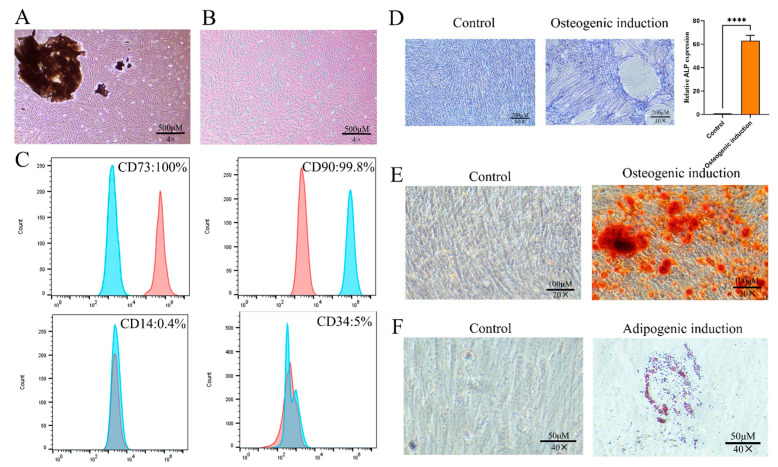
Culture and identification of PDLSCs. (**A**) Primary PDLSCs. Scale bar = 500 µm. (**B**) P1 PDLSCs. Scale bar = 500 µm. (**C**) Expression of CD73, CD90, CD14, and CD34 in PDLSCs. (**D**) Alkaline phosphatase (ALP) staining of PDLSCs and quantitative analysis for osteogenic differentiation. Scale bar = 200 µm. (**E**) Alizarin red staining of PDLSCs for osteogenic differentiation. Scale bar = 100 µm. (**F**) Oil red O staining of PDLSCs for adipogenic differentiation. Scale bar = 50 µm. **** *p* < 0.0001.

The isolated periodontal ligament tissue explants exhibited successful adherence to the culture flask with no observable abnormalities in the medium. Primary (P0) PDLSCs displayed a characteristic long spindle-shaped morphology (Figure 1A). Upon passaging, the P1 cells maintained a homogeneous fibroblast-like appearance (Figure 1B). Flow cytometric analysis confirmed the mesenchymal stem cell phenotype of the cultured cells, demonstrating a positive expression of CD73 and CD90, while being negative for CD14 and CD34 (Figure 1C), which is consistent with the established PDLSC markers. To assess the multipotent differentiation capacity of the isolated cells, osteogenic and adipogenic induction were performed. We performed alkaline phosphatase (ALP) staining after 7 days of osteogenic induction, showing a significant increase in ALP activity (Figure 1D). By day 21, we observed the clear formation of mineralized nodules through Alizarin Red staining (Figure 1E). Similarly, after 21 days of adipogenic induction, we detected evident lipid droplet accumulation via Oil Red O staining (Figure 1F). These findings collectively confirm the successful isolation and characterization of PDLSCs with typical stem cell properties.

### 2.2. Characterization of GelMA Hydrogel

**Figure 2:**
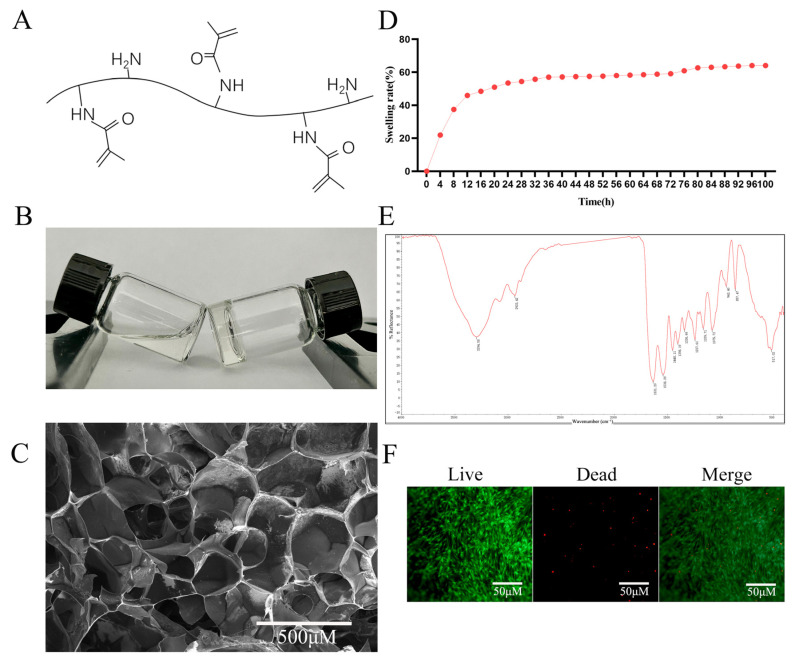
Characterization of the GelMA hydrogel. (**A**) Molecular structure of GelMA. (**B**) Photograph of GelMA. (**C**) FESEM image of the cross-section of GelMA. Scale bar = 500 µm. (**D**) Swelling ratio of GelMA. (**E**) FTIR analysis of GelMA. (**F**) Cytotoxicity test of GelMA hydrogel on PDLSCs. Scale bar = 50 µm.

The synthesized GelMA hydrogel was systematically characterized through multiple analytical approaches. The molecular structure of the GelMA hydrogel is shown in Figure 2A. Rheological assessment demonstrated a distinct phase transition, where uncured GelMA maintained fluidity (left vial in Figure 2B), while photopolymerized GelMA formed a stable network losing its flowability (right vial in Figure 2B). FESEM examination revealed an interconnected porous architecture with an average pore diameter of 100–200 μm, as quantified by ImageJ (v1.53q) analysis (Figure 2C), which is ideal for MSCs infiltration and nutrient diffusion. Swelling kinetics analysis of GelMA showed rapid water uptake during the initial 12 h, achieving equilibrium swelling at 24 h with a swelling ratio of 53.5% (Figure 2D). This phenomenon demonstrates the material’s excellent hydrophilic properties. The swelling of hydrogels results in enhanced permeability and nutrient diffusion, making them a potential drug delivery material [22]. The FTIR spectrum displays a series of characteristic peaks that correspond to the vibrational modes of various chemical bonds within the GelMA molecule (Figure 2E). According to the spectrum, the C = C vibration originating from the newly introduced methacrylate double bond appears at 1631.20 cm^−1^, as reported in analogous synthetic studies [23]. Most importantly, live/dead staining after 72 h of culture showed predominant green fluorescence (viable cells) with minimal red fluorescence (dead cells), yielding a cell viability >95% (Figure 2F). This excellent cytocompatibility, combined with its optimal physical properties, establishes GelMA as a suitable scaffold for drug delivery applications.

### 2.3. Determination of Optimal Concentrations for GelMA@G-Rb3 and LPS Treatment

**Figure 3:**
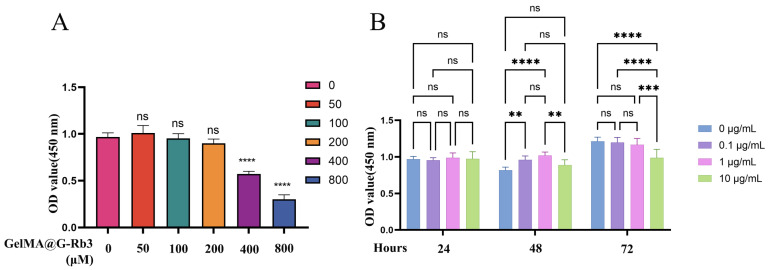
Determination of safe concentrations of G-Rb3 and LPS. (**A**) Cytotoxicity of different concentrations of G-Rb3 on PDLSCs. (**B**) Cytotoxicity of different concentrations of LPS on PDLSCs. ** *p* < 0.01, *** *p* < 0.001, **** *p* < 0.0001, ns: no significant.

The CCK-8 assay revealed the concentration-dependent effects of GelMA@G-Rb3 on PDLSC proliferation after 52 h of culture (Figure 3A). Concentrations of 50–200 µM maintained normal cell viability, while higher concentrations, i.e., 400 µM and 800 µM, significantly inhibited the proliferation of PDLSCs. Time-course evaluation of LPS effects demonstrated that all tested concentrations (0.1–10 µg/mL) showed comparable proliferation rates at 24 h. By 48 h, LPS at 0.1–1 μg/mL significantly enhanced proliferation, whereas 10 μg/mL exhibited neutral effects. At 72 h, significant inhibition was observed exclusively with 10 μg/mL LPS (Figure 3B). Based on these findings, 100 µM and 200 µM GelMA@G-Rb3 and 1 µg/mL LPS were selected as the optimal concentration for subsequent experiments. PDLSCs were pretreated with GelMA@G-Rb3 at concentrations of 100 or 200 μM, selected to maximize therapeutic efficacy while minimizing cytotoxicity, for 2 h prior to LPS (1 μg/mL) stimulation, and subsequently co-cultured for 24 h for qRT-PCR analysis or 48 h for Western blot and ELISA detection to coincide with peak gene expression and peak protein expression, respectively.

### 2.4. Anti-Inflammatory Effects of GelMA@G-Rb3 on LPS-Stimulated PDLSCs

**Figure 4:**
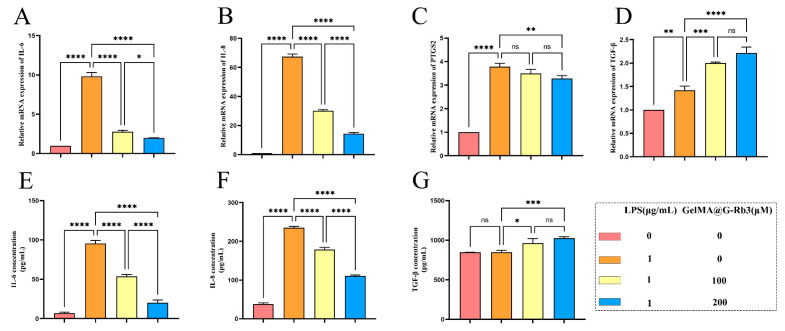
Effects of GelMA@G-Rb3 on the expression of inflammation-related genes and proteins in LPS-stimulated PDLSCs. (**A**–**D**) Expression of IL-6 (**A**), IL-8 (**B**), PTGS2 (**C**), and TGF-β (**D**) mRNA in LPS-stimulated PDLSCs co-cultured with GelMA@G-Rb3 for 24 h. (**E**–**G**) The expression of IL-6 (**E**), IL-8 (**F**), and TGF-β (**G**) in the supernatant of GelMA@G-Rb3 and LPS-stimulated PDLSCs after co-culture for 48 h was detected by ELISA. * *p* < 0.05, ** *p* < 0.01, *** *p* < 0.001, **** *p* < 0.0001, ns: no significant.

qRT-PCR analysis demonstrated a significant modulation of inflammatory responses by GelMA@ G-Rb3 (Figure 4A–D). The LPS challenge markedly up-regulated pro-inflammatory cytokines IL-6, IL-8, and PTGS2, confirming successful inflammatory model establishment. At concentrations of 100 μM and 200 μM, the expression of IL-6 was reduced by 71.78% and 79.62%, respectively; IL-8 decreased by 55.15% and 78.72%, respectively; PTGS2 declined by 7.74% and 13.54%, respectively; while TGF-β increased by 1.4-fold and 1.6-fold, respectively. Notably, both 100 µM and 200 µM GelMA@G-Rb3 pretreatment effectively suppressed these inflammatory mediators in a concentration-dependent manner, with 200 µM showing superior inhibition efficacy. Conversely, the expression profiles of TGF-β, an important anti-inflammatory factor, exhibited a dose-responsive elevation following GelMA@G-Rb3 treatment, suggesting concurrent immune modulation.

Consistent with transcriptional findings, ELISA analysis showed that 48 h LPS stimulation significantly increased IL-6 and IL-8 secretion (Figure 4E,F). GelMA@G-Rb3 pretreatment at 100 µM and 200 µM attenuated this response dose-dependently, achieving 43.9% and 78.7% reduction in IL-6, and 24.1% and 52.8% in IL-8, respectively (Figure 4E,F). Parallel to qRT-PCR results, TGF-β production was enhanced by 1.8- and 2.3-fold in 100 µM and 200 µM treatment groups (Figure 4G).

### 2.5. Network Pharmacology Analysis and Mechanistic Investigation of GelMA@G-Rb3 in Periodontitis Treatment

**Figure 5:**
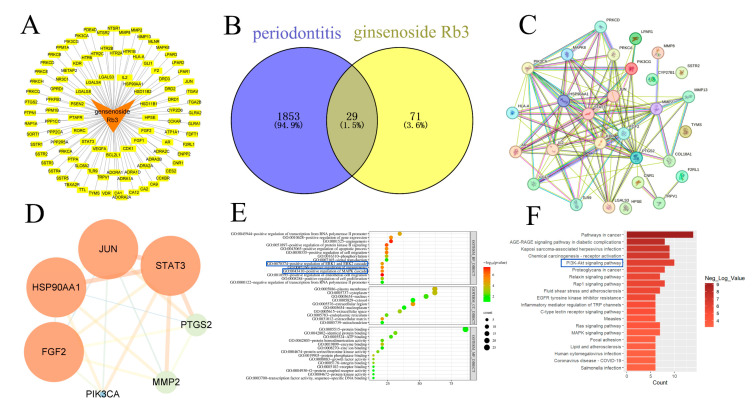
Network pharmacology analysis of G-Rb3 in the treatment of periodontitis. (**A**) Network relationship diagram of G-Rb3 and its targets. (**B**) Intersection targets of G-Rb3 and periodontitis. (**C**) Protein–protein interaction (PPI) network of G-Rb3 in periodontitis. (**D**) Core target network of G-Rb3 in periodontitis. (**E**) Results of GO enrichment analysis. (**F**) Results of KEGG pathway analysis.

Comprehensive target screening identified 100 potential targets of G-Rb3 through the Swiss Target Prediction database, which were used to construct a target network (Figure 5A). Disease-related targets were systematically collected from multiple databases, yielding 1882 unique periodontitis-associated targets after merging and deduplication (GeneCards: 1609; OMIM: 8; DisGeNET: 682). Venn diagram analysis revealed 29 overlapping targets between G- Rb3 and periodontitis, including key mediators such as matrix metalloproteinase 2 (MMP2), MMP8, and PTGS2 (Figure 5B). PPI network analysis via the STRING platform (Figure 5C) and subsequent Cytoscape (v3.9.1) visualization (Figure 5D) identified core therapeutic targets. Functional GO enrichment analysis demonstrated that these targets were significantly associated with critical biological processes, including the following: positive regulation of RNA polymerase II promoter transcription, modulation of gene expression, angiogenesis, and the regulation of ERK1/2 and MAPK signaling cascades (Figure 5E). KEGG pathway analysis highlighted involvement in several key pathways including cancer-related pathways, advanced glycation end products-receptor for advanced glycation end products (AGE-RAGE) signaling in diabetic complications, and PI3K-Akt signaling pathway (Figure 5F). Previous studies have indicated that the AGE-RAGE and PI3K-Akt signaling pathways play significant roles in the pathogenesis of periodontitis [24,25]. Elevated serum CRP levels in periodontitis patients impair insulin signaling, promoting hyperglycemia and the subsequent accumulation of advanced glycation end products (AGEs) in periodontal tissues. These AGEs engage RAGE receptors on immune cells, triggering pro-inflammatory cytokine release that directly drives periodontal destruction and osteoclastogenesis. Additionally, AGE-RAGE interactions dysregulate innate immunity by inducing the hyperactivation or functional suppression of macrophages and neutrophils, compromising pathogen clearance. This signaling pathway further exacerbates disease progression by promoting the colonization of Gram-negative anaerobic bacteria, establishing a vicious cycle of inflammation and tissue breakdown. In periodontitis, PI3K-Akt signaling is activated by keystone pathogens such as Porphyromonas gingivalis and serves as a central hub that suppresses the intrinsic apoptosis of gingival epithelial and immune cells, thereby prolonging bacterial persistence within the tissue. Sustained Akt activity amplifies the inflammatory milieu by coupling to the NF-κB–dependent transcription of IL-6, TNF-α, and other pro-inflammatory mediators that drive connective-tissue destruction and alveolar bone resorption. Beyond local tissue damage, chronic PI3K-Akt activation facilitates oncogenic reprogramming: it downregulates pro-apoptotic proteins, induces epithelial–mesenchymal transition through the modulation of GSK3β and Zeb1, and cooperates with JAK/STAT and MAPK cascades to promote cell proliferation, migration, and metastasis, thus linking periodontal infection to increased risk of orodigestive cancers.

**Figure 6:**
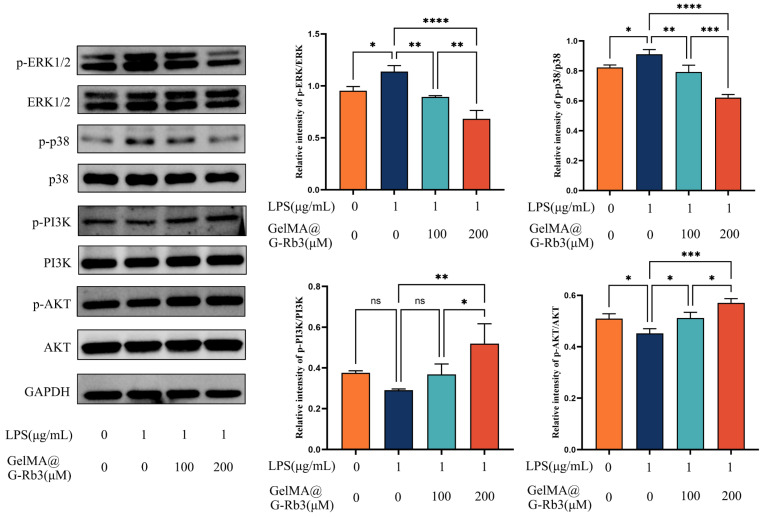
Involvement of signaling pathways in LPS-stimulated PDLSCs by GelMA@G-Rb3. After the co-culture of LPS-stimulated PDLSCs with GelMA@G-Rb3 for 48 h, the protein expression levels of p-ERK1/2, ERK1/2, p-p38, p38, p-PI3K, PI3K, p-AKT, AKT, and GAPDH were shown. Statistical analysis of p-ERK/ERK, p-p38/p38, p-PI3K/PI3K, and p-AKT/AKT ratios was also performed. * *p* < 0.05, ** *p* < 0.01, *** *p* < 0.001, **** *p* < 0.0001, ns: no significant.

Building upon the network pharmacology predictions, we experimentally validated the anti-inflammatory mechanisms of GelMA@G-Rb3 in LPS-challenged PDLSCs. The WB result demonstrated that LPS stimulation markedly increased the phosphorylation of p38 and ERK1/2 and GelMA@G-Rb3 treatment dose-dependently suppressed this LPS-induced phosphorylation. LPS stimulation significantly suppressed both PI3K and Akt phosphorylation. Treatment with 100 μM GelMA@G-Rb3 partially restored PI3K phosphorylation and markedly increased Akt phosphorylation, while 200 μM GelMA@G-Rb3 further enhanced PI3K phosphorylation to significant levels and induced a more pronounced upregulation of Akt phosphorylation. (Figure 6). These results collectively demonstrated that GelMA@G-Rb3 exerts its therapeutic effects through the dual modulation of MAPK and PI3K/Akt signaling pathways, aligning with the predicted targets from network pharmacology analysis.

### 2.6. Evaluation of GelMA@G-Rb3 Biocompatibility and Periodontal Regeneration Capacity

To evaluate the in vivo biocompatibility of GelMA@G-Rb3, histological examination was performed on major organs (liver, spleen and lungs) using H&E staining. The results demonstrated well-preserved tissue architecture in all treatment groups without observable signs of toxicity or pathological alterations (Figure S1), confirming the material’s safety profile for in vivo applications.

To systematically evaluate the anti-inflammatory efficacy and tissue regeneration potential of GelMA@G-Rb3, we conducted comprehensive validation using micro-CT scanning and histopathological analysis in the present study.

**Figure 7:**
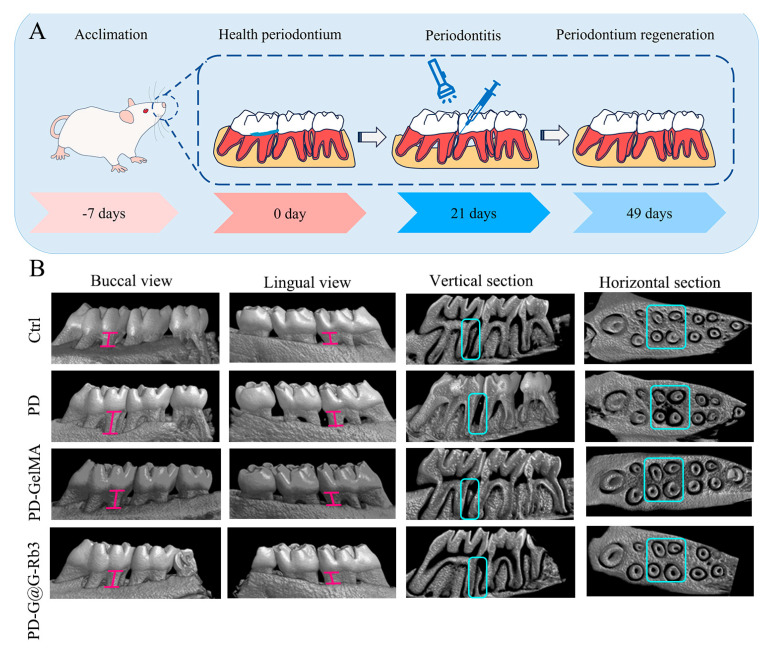
Effect of GelMA@G-Rb3 on in situ bone formation in rat periodontitis models. (**A**) Flowchart of GelMA@G-Rb3-based procedure to treat periodontitis. (**B**) The micro-CT scanning results showed that significant alveolar bone resorption was shown in the periodontitis group (PD). After the treatment with GelMA@G-Rb3(PD-G@G-Rb3), the height of the alveolar bone increased significantly, which was significantly higher than that with GelMA treatment only (PD-GelMA) and was recovered to almost normal levels in control group (Ctrl). The pink I-shaped marker indicates the distance from the enamel-cementum junction to the alveolar crest. .H&E staining of periodontal tissues (Figure 8A) demonstrated severe periodontal destruction in the periodontitis group (PD), featuring disrupted gingival papilla architecture, pronounced attachment loss, dense and inflammatory cell infiltration. Moderate improvement was observed in the GelMA-only group and significant therapeutic effects were observed in the GelMA@G-Rb3 group, as shown by preserved tissue attachment and reduced inflammatory infiltration.

The regeneration of alveolar bone was examined by micro-CT analysis (Figure 7B). The micro-CT scanning results showed that compared with the control group (Ctrl), the periodontitis group (PD) exhibited significant alveolar bone resorption in both vertical and horizontal sections of the alveolar bone. Conversely, after the treatment with GelMA@G-Rb3 (PD-G@G-Rb3), the height of the alveolar bone increased significantly, which was significantly higher than that of GelMA treatment only (PD-GelMA) and was recovered to almost normal levels in the control group (Ctrl).

**Figure 8:**
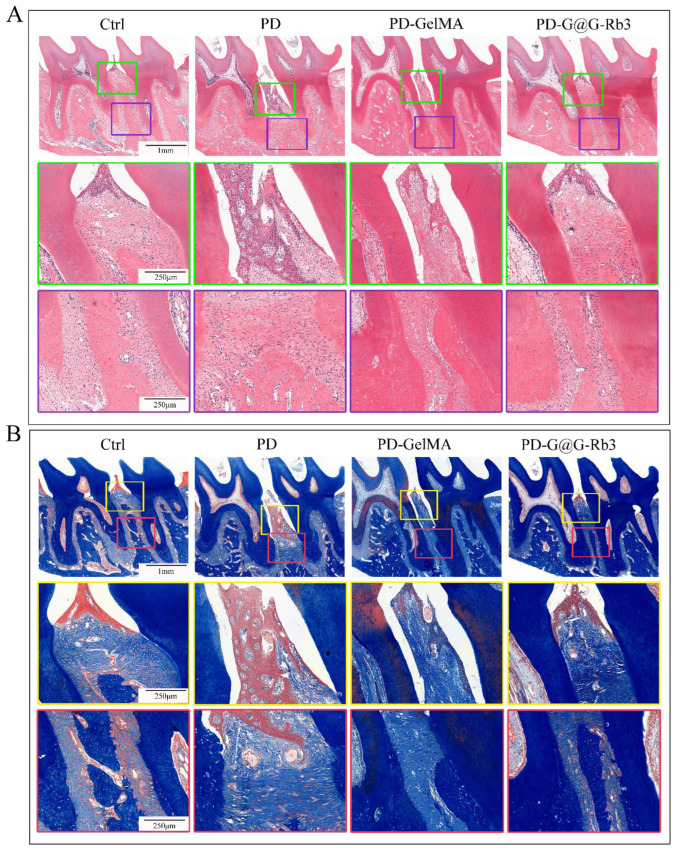
Histological staining of the periodontal tissues in rats. (**A**) H&E staining. The scale bars in the upper row, middle row, and lower row are 1 mm, 250 µm, and 250 µm, respectively. (**B**) Masson’s trichrome staining. The scale bars in the upper row, middle row, and lower row are 1 mm, 250 µm, and 250 µm, respectively.

Masson’s trichrome staining (Figure 8B) revealed pathological changes in the periodontitis group (PD) such as fragmented collagen fibers, disrupted periodontal ligament, and advanced alveolar bone resorption. Partial amelioration was observed with GelMA treatment and superior tissue restoration was observed with GelMA@G-Rb3, such as a well-organized collagen matrix, regenerated periodontal ligament, and preserved alveolar bone structure.

**Figure 9:**
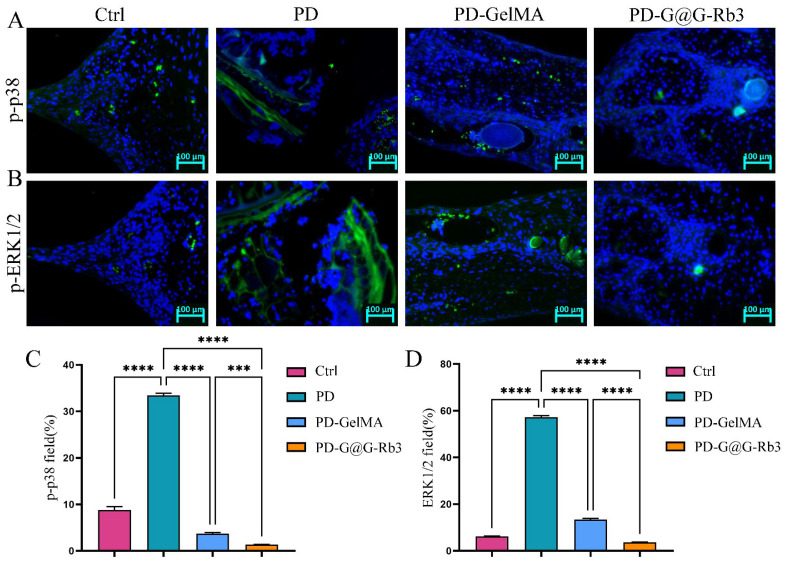
Immunofluorescence staining of periodontal tissue in rats. (**A**,**C**) Immunofluorescence staining of p-p38. Scale bar = 100 µm. (**B**,**D**) Immunofluorescence staining of p-ERK1/2. Scale bar = 100 µm. *** *p* < 0.001, **** *p* < 0.0001, ns: no significant.

Subsequently, immunofluorescence staining was performed to further validate the regulatory effects of GelMA@G-Rb3 on key signaling pathways. Immunofluorescence analysis (Figure 9) revealed distinct expression patterns of p-p38 (Figure 9A,C) and p-ERK1/2 (Figure 9B,D) across treatment groups. The control group exhibited baseline expression with weak fluorescence signals, while the periodontitis group (PD) demonstrated marked pathway hyper-activation characterized by strong fluorescence intensity and numerous positive cells. The GelMA group showed intermediate pathway inhibition, whereas the GelMA@G-Rb3 treatment group displayed the most pronounced therapeutic effect, with markedly reduced fluorescence signals, few positive cells, and effective suppression of inflammatory signaling pathway activation. However, the current study focused specifically on detecting key nodal proteins in the MAPK pathway, while the analysis of PI3K/AKT signaling will be incorporated as part of our ongoing research program.

The prevention and treatment of periodontitis can be approached from two main aspects, one is by alleviating bacterial virulence through the removal of periodontal bacteria or the use of antimicrobial therapies, and the other is by blocking the destruction of periodontal tissues caused by bioactive substances, such as cytokines, prostaglandins, and metalloproteinases produced during the host response, thereby modulating the host’s defense mechanisms or enhancing the body’s resistance. In clinical practice, periodontal initial therapy, including supragingival scaling, subgingival scaling, and root planing, are the primary methods for removing plaque and controlling infection and inflammation. However, due to the complexity of anatomical structures such as periodontal pockets or root furcations, these mechanical treatments alone often fail to achieve ideal results [26], necessitating novel treatment strategies offering an optimal therapeutic approach to enhance efficacy. In recent years, the application of local or systemic antibiotics has been considered an effective means to enhance the outcomes of periodontal treatment. However, the misuse of antibiotics may lead to increased bacterial resistance [27], prompting the restriction of systemic antibiotics to specific cases [28]. Additionally, while local antibiotics have demonstrated certain clinical efficacy, limitations such as an insufficient antimicrobial spectrum, risk of resistance, and high costs [28] have hindered their widespread use.

Meanwhile, G-Rb3, a natural compound with remarkable anti-inflammatory properties, could effectively inhibit the release of inflammatory mediators through multiple mechanisms [29,30,31], demonstrating broad and diverse anti-inflammatory effects. However, its systemic administration faces numerous challenges, including issues of drug resistance, difficulty in reaching the infection site, failure to achieve effective concentrations, potential gastrointestinal adverse reactions, and inadequate tissue permeability, which limit its application in the treatment of periodontitis.

These limitations have driven researchers to turn their attention to local drug delivery systems. By directly placing drugs subgingivally or into periodontal pockets, local delivery systems enable sustained or controlled drug release while minimizing systemic adverse effects on patients [32]. Hydrogel-based drug delivery systems, leveraging their unique structure and properties, are able to precisely fill periodontal pockets through injection and form stable drug reservoirs locally to ensure sustained drug release. Their excellent biocompatibility, biodegradability, and superior drug release performance make them safe, practical, and effective for periodontitis treatment [33]. As a result, hydrogel-based drug delivery systems offer a novel approach and broad prospects for the local treatment of periodontitis.

GelMA hydrogel has been widely used in various tissue engineering and regenerative medicine fields due to its excellent biocompatibility and adjustable physicochemical properties. In this experiment, we successfully prepared the GelMA hydrogel, and verified its consistency with standard GelMA hydrogel characteristics using FTIR and FESEM. The hydrogel prepared in this experiment exhibited excellent swelling properties, which is a key indicator for evaluating the performance of hydrogels as drug delivery systems. Moreover, through live/dead cell staining experiments, we further confirmed that GelMA@G-Rb3 possesses outstanding biocompatibility. Our findings align with prior reports on GelMA’s biocompatibility [16,17] but extend its application by demonstrating synergistic effects with G-Rb3. Unlike conventional antibiotics, GelMA@G-Rb3 avoids systemic side effects while achieving sustained local release. Moreover, the dual inhibition of P38/ERK and activation of PI3K/AKT mirrors the multi-target anti-inflammatory actions of ginsenosides, yet our study uniquely couples this with hydrogel-mediated spatial control, offering a blueprint for precision medicine in periodontitis.

The injectability and photocrosslinking properties of GelMA enable minimally invasive delivery and in situ gelation, effectively addressing the anatomical challenges posed by periodontal pockets. However, previous studies have demonstrated the localized delivery of antibiotics and cytokines to gingival tissues for immunomodulation and tissue regeneration [34]. Additionally, innovative approaches combining biotin-avidin systems with GelMA have been employed to deliver mesenchymal stem cell-derived exosomes, promoting the regeneration of defective periodontal tissues [35]. These therapeutic strategies provide valuable insights for our research direction, suggesting that the dual functionality of GelMA as both a drug carrier and tissue-regenerative scaffold could offer superior therapeutic outcomes for periodontitis treatment.

As a chemokine and pleiotropic cytokine, respectively, IL-8 and IL-6 play an important role in inflammation and immune responses. And, studies have shown that the levels of TGF-β1 in the serum, saliva, and gingival crevicular fluid of periodontitis patients are significantly elevated [36]. It was shown that the levels of IL-8 and IL-6 in the saliva of patients with periodontitis gradually increase over time, while these inflammatory factor levels significantly decrease after non-surgical clinical treatment [37]. Based on these findings, we selected IL-6, IL-8, TGF-β, and PTGS2, which are determined from network pharmacology analysis, as research targets. The results showed that after the LPS stimulation of PDLSCs, the gene expression levels of IL-6, IL-8, and PTGS2 significantly increased, while the GelMA@G-Rb3 treatment effectively reduced the expression levels of these inflammatory factors. Moreover, GelMA@G-Rb3 significantly increased the expression level of TGF-β, with similar trends observed at the protein level.

To elucidate the regulatory mechanism of GelMA@G-Rb3 on inflammatory responses in periodontitis, we employed network pharmacology methods to systematically analyze the potential targets of G-Rb3 in periodontitis treatment. Network pharmacology analysis revealed that G-Rb3 may exert its effects by modulating multiple signaling pathways, including MAPK and PI3K-AKT pathways, which were further investigated using Western blot analysis. The experimental data indicated that GelMA@G-Rb3 effectively inhibits the activation of the MAPK signaling pathway, thereby alleviating LPS-induced inflammatory responses. At the same time, GelMA@G-Rb3 activates the PI3K-AKT signaling pathway, and reduces the inflammatory effects caused by LPS. These findings align with previous studies on these pathways [38,39,40,41,42,43,44], providing molecular mechanism support for the potential application of G-Rb3 in the treatment of periodontitis.

The in vitro experiments of this study have confirmed that GelMA@G-Rb3 exhibits significant anti-inflammatory effects on LPS-stimulated PDLSCs. Based on these findings, we further conducted in vivo experiments to validate the specific effects of GelMA@G-Rb3 on periodontal tissues in rats with periodontitis. After successfully establishing a rat periodontitis model, we injected GelMA@G-Rb3 directly into the periodontal pockets using a syringe and rapidly performed photopolymerization. Following the treatment, we observed the obvious regenerative effects of GelMA@G-Rb3 on the defects of periodontal tissues of rats with periodontitis. Micro-CT three-dimensional imaging, H&E staining, and Masson staining results further confirmed that GelMA@G-Rb3 effectively promotes the regeneration of periodontal tissues in rats with periodontitis.

However, this study has some limitations that should be addressed. First, while we identified the involvement of the MAPK/PI3K-AKT pathway, the precise molecular mechanisms and downstream effectors require further elucidation. Second, the rodent model used in this study, though widely adopted for preliminary investigations, presents anatomical and immunological differences from human periodontium. Future studies should employ large animal models (e.g., porcine or non-human primates) that better mimic human periodontal structures and disease progression before clinical translation.

## 3. Conclusions

In this study, an injectable photopolymerizable GelMA@G-Rb3 composite hydrogel was developed. In vitro experiments demonstrated that GelMA@G-Rb3 effectively regulates the inflammatory response in periodontitis by modulating the MAPK/PI3K-AKT signaling pathway, significantly suppressing the expression of pro-inflammatory factors while promoting the production of anti-inflammatory factors. While in vivo analysis confirmed GelMA@G-Rb3’s remarkable anti-inflammatory effects, including reduced periodontal inflammation, decreased alveolar bone resorption, promoted tissue regeneration, and significant downregulation of MAPK signaling, future studies will expand pathway validation to fully characterize the PI3K-AKT contribution in vivo. These findings establish GelMA@G-Rb3 as a promising therapeutic strategy for periodontitis, providing both theoretical and experimental foundations for novel periodontal treatments.

## 4. Materials and Methods

### 4.1. Isolation and Culture of PDLSCs

This study was approved by the Medical Ethics Committee of Shandong Second Medical University (No. 2024YX057) and conducted in accordance with the Declaration of Helsinki. PDLSCs were isolated from 22 surgically extracted impacted third molars obtained from healthy donors aged 18–23 years who provided written informed consent. All teeth were carefully selected through clinical examination based on strict criteria: absence of caries, periodontal disease (probing depth ≤ 3 mm), and any systemic conditions in the donors. Under sterile conditions, periodontal ligament tissues from the root middle third were minced into approximately 1 mm^3^ fragments and cultured in MEM-α medium (Yuanpei Biotechnology, Shanghai, China) supplemented with 20% fetal bovine serum (FBS; Gibco, Gaithersburg, MA, USA), 100 U/mL penicillin, and 100 μg/mL streptomycin (both from Biosharp, Shanghai, China). Cells were maintained at 37 °C in a humidified atmosphere containing 5% CO_2_. When reaching 80–90% confluence, cells were subcultured using standard protocols, and passages 3–5 were used for subsequent experiments.

### 4.2. Characterization and Differentiation of PDLSCs

According to the ISCT criteria, MSCs must express positive markers such as CD73 and CD90, and must not express negative markers including CD14 and CD34 [45]. Therefore, when identifying PDLSCs (a subset of MSCs), CD73 and CD90 are selected as positive markers, while CD14 and CD34 serve as negative markers, ensuring that the characterized cells meet the fundamental features of MSCs. PDLSCs were characterized by flow cytometry following standard protocols. Cells were harvested using 0.25% trypsin-EDTA (Gibco) and stained with the fluorochrome-conjugated antibodies against CD14-FITC, CD34-APC, CD73-PE, and CD90-FITC (all from BioLegend, San Diego, CA, USA). Appropriate isotype-matched controls were included for each marker. Cell surface marker expression was analyzed using a BD FACSCanto II flow cytometer (BD Biosciences, Franklin Lakes, NJ, USA), with data processed using FlowJo software (v10.8.1, LLC, Ashland, OR, USA). For osteogenic differentiation, cells were seeded at a density of 1.2 × 10^5^ cells/well in 6-well plates and cultured in osteogenic medium consisting of MEM-α (Yuanpei Biotechnology) supplemented with 10% FBS (Gibco), 10 nM dexamethasone (Solarbio, Beijing, China), 10 mM β-glycerophosphate (Solarbio), and 50 μg/mL ascorbic acid (Solarbio). The medium was replaced every 3 days for 21 days. Differentiation was assessed by ALP activity at day 7 using BCIP/NBT staining (Beyotime Biotechnology) and calcium deposition at day 21 using 2% Alizarin Red S (pH 4.2; Sigma-Aldrich, Saint Louis, MO, USA) staining. Adipogenic differentiation was induced using a medium supplemented with 10 μM dexamethasone, 200 μM indomethacin, 10 μg/mL insulin, and 0.5 mM 3-isobutyl-1-methylxanthine (Sigma-Aldrich) for 21 days, with lipid accumulation visualized by Oil Red O staining (Solarbio). All differentiation assays included fixation with 4% paraformaldehyde prior to staining, and results were documented using inverted microscopy (Nexcope, Ningbo, China).

### 4.3. Synthesis and Characterization of GelMA Hydrogel

The GelMA hydrogel was prepared by dissolving GelMA powder (EFL, Suzhou, China) in phosphate-buffered saline (PBS) containing 0.25% (*w*/*v*) lithium phenyl-2, 4, 6- trimethylbenzoylphosphinate as a photoinitiator. The solution was incubated at 60–70 °C for 30 min with periodic agitation to ensure complete dissolution, followed by sterile filtration through a 0.22 μm sterile syringe filter (Yeasen, Shanghai, China). Prior to photocrosslinking with a 405 nm visible light (25 mW/cm^2^, 20 s), the 5% (*w*/*v*) GelMA precursor solution was injected into a cylindrical through-hole PDMS mold (custom-made, MicroX Medical Technology, Taizhou, China) positioned on a release film (EFL, Suzhou, China) covering the desktop light-curing platform (EFL, Suzhou, China), where it solidified into three-dimensional hydrogels.

Morphological analysis. Lyophilized hydrogel samples were sputter-coated with gold and examined by an Apreo field emission scanning electron microscope (FESEM; Thermo Fisher Scientific, Waltham, MA, USA) to evaluate their microarchitecture and porosity.

Swelling behavior. The equilibrium swelling ratio (SR) was determined by measuring mass changes (*n* = 3) after immersion in PBS at 37 °C. The SR was calculated using the formula: SR (%) = [(m_t_ − m_0_)/m_0_] × 100%, where m_0_ and m_t_ represent the initial and time-dependent wet weights, respectively.

Chemical characterization. Nicolet iS50 FTIR Spectrometer (Thermo Fisher Scientific, Waltham, MA, USA) was performed on freeze-dried samples in the range of 500–4000 cm^−1^ to verify the chemical structure and successful methacrylation.

Rheological properties. The injectability of the pre-gel solution was qualitatively assessed by observing its flow behavior during vial tilting tests before and after photocrosslinking.

### 4.4. Biocompatibility and Cytotoxicity Evaluation of GelMA and LPS on PDLSCs

To assess the biocompatibility of GelMA hydrogel and determine safe concentrations of GelMA@G-Rb3 and LPS, we performed live/dead cell staining and Cell Counting Kit-8 (CCK-8) proliferation assays. For live/dead evaluation, PDLSCs (3 × 10^4^ cells/well) were cultured on GelMA hydrogels (80 µL/well) for 96 h, followed by staining with Calcein AM/PI (Beyotime, Shanghai, China) and fluorescence microscopy (Zeiss, Oberkochen, Germany) analysis. CCK-8 assays were conducted to establish optimal concentrations. GelMA@G-Rb3 (50–800 µM) and LPS (0.1–10 µg/mL) were tested on PDLSCs seeded in 96-well plates (5 × 10^3^ cells/well). After various treatment periods, i.e., 24–72 h for LPS and 52 h for GelMA@G-Rb3, respectively, cell viability was measured by absorbance at 450 nm following CCK-8 reagent (Dojindo, Kumamoto, Japan) incubation, with six replicates for each concentration.

### 4.5. Quantitative Real-Time Polymerase Chain Reaction (qRT-PCR) Analysis

Total RNA was extracted from treated PDLSCs using a TRIzol reagent (Accurate Biotechnology (Hunan) Co., Ltd., Changsha, China) according to the manufacturer’s protocol. RNA concentration and purity were determined spectrophotometrically (NanoReady, Life Real, Hangzhou, China), with A260/A280 ratios between 1.8 and 2.0 indicating high-quality RNA. Subsequently, 1 μg of total RNA was reverse-transcribed using the EVo M-MLV RT Mix Kit with gDNA Clean for qPCR Ver.2 (Accurate Biotechnology). Quantitative PCR was performed using a 20 μL reaction system containing 2 × SYBR Green Pro Taq HS Premix (Accurate Biotechnology) and gene-specific primers (Sangon Biotech, Shanghai, China) for target genes. Primers sequences, including interleukin (IL)-6, IL-8, prostaglandin-endoperoxide synthase 2 (PTGS2), and transforming growth factor-β (TGF-β) and the reference gene GAPDH, were listed in Table 1. Reactions were carried out in triplicate on a Bio-Rad CFX96 Real-Time PCR System (Bioer technology, Hangzhou, China) under the following cycling conditions: initial denaturation at 95 °C for 30 s, followed by 40 cycles of 95 °C for 5 s and 60 °C for 30 s. Melting curve analysis was performed to verify amplification specificity. Relative gene expression levels were calculated using the 2^−ΔΔCt^ method and normalized to GAPDH as an internal control.

### 4.6. Enzyme-Linked Immunosorbent Assay (ELISA) Analysis

The concentrations of IL-6, IL-8, and TGF-β in cell culture supernatants collected from the lower chamber of transwell plates were quantified using commercial ELISA kits (Neobioscience, Shenzhen, China) according to the manufacturer’s protocols. Briefly, 100 μL of each supernatant sample was added to antibody-coated wells and incubated for 2 h at 37 °C. After washing, horseradish peroxidase (HRP)-conjugated detection antibodies were added and incubated for 1 h at 37 °C. Following additional washes, 3,3′,5,5′-tetramethylbenzidine substrate solution was added, and the enzymatic reaction was stopped after 15 min with 2 M sulfuric acid. The optical density at 450 nm was measured using a multifunctional microplate reader (SuPerMax 3100, Shanghai, China), with a reference wavelength set at 630 nm. All samples were assayed in triplicate, and cytokine concentrations were calculated against standard curves generated for each target protein.

### 4.7. Network Pharmacology Analysis

The network pharmacology analysis was systematically performed by first identifying G-Rb3-related targets through TCMSP (https://tcmsp-e.com/, accessed on 23 May 2024), PubChem (https://pubchem.ncbi.nlm.nih.gov/, accessed on 23 May 2024), and Swiss Target Prediction (http://old.swisstargetprediction.ch/, accessed on 23 May 2024) databases, while periodontitis-associated targets were retrieved from GeneCards (https://www.genecards.org/, accessed on 23 May 2024), OMIM (https://omim.org/, accessed on 23 May 2024), and DisGeNET (https://www.disgenet.org/, accessed on 23 May 2024) databases. After merging and deduplicating the datasets, compound-target relationships were visualized using Cytoscape software (v3.9.1; https://cytoscape.org/, accessed on 23 May 2024). Common targets were identified via VENNY 2.1 (https://bioinfogp.cnb.csic.es/tools/venny/, accessed on 23 May 2024), followed by protein–protein interaction (PPI) network construction using a STRING database (v11.5, confidence score > 0.7; https://string-db.org/, accessed on 24 May 2024) and core target screening with a CytoHubba plugin. Finally, GO and KEGG enrichment analyses were conducted using DAVID (v6.8, *p* < 0.05; https://david.ncifcrf.gov/, accessed on 25 May 2024), with results visualized using bioinformatics online tools (https://www.bioinformatics.com.cn/plot_basic_gopathway_enrichment_bubbleplot_081, accessed on 25 May 2024) and R programming (v4.4.2).

### 4.8. Western Blot Analysis

For protein extraction, cells were washed with ice-cold PBS and lysed in radioimmunoprecipitation assay buffer (Solarbio) containing 1 mM phenylmethylsulfonyl fluoride and protease/phosphatase inhibitors (Solarbio) on ice for 30 min. After centrifugation (12,000 rpm, 15 min, 4 °C), protein concentrations were determined by bicinchoninic acid assay. Equal amounts of protein (20 μg) were mixed with 4× loading buffer (Solarbio), denatured (100 °C, 5 min), separated by 10% SDS-PAGE (Biosharp), and transferred to methanol-activated PVDF membranes (Merck Millipore, Burlington, MA, USA). Membranes were blocked with rapid blocking buffer (Affinity LifeScience, Wuhan, China) for 10 min at room temperature, then incubated with primary antibodies (Cell Signaling Technology, Danvers, MA, USA) against ERK1/2 (1:1000), p-ERK1/2 (1:2000), p38 (1:1000), p-p38 (1:1000), anti-rabbit IgG (1:10,000), protein kinase B (Akt; 1:2000), p-Akt (1:2000), phosphatidylinositol 3-kinase (PI3K; 1:1000), p-PI3K (1:1000), and GAPDH (1:10,000; Bioprimacy, Hubei, China) at 4 °C overnight, followed by incubation with the HRP-conjugated secondary antibody (1:10,000) for 1 h at room temperature. Bands were detected using the ECL reagent (Biosharp) and imaged with a ChemiDoc MP system (Bio-Rad, Hercules, CA, USA).

### 4.9. Periodontitis Model Establishment and Treatment in Rats

Male specific pathogen free-grade Sprague-Dawley rats (4 weeks old, 180–220 g; Pengyue Animal Breeding Center, Jinan, China) were acclimatized for 1 week before experimentation. Animal welfare and experimental procedures were carried out in accordance with the protocols approved by the Animal Ethics Committee of Shandong Second Medical University (2024SDL627). As shown in Figure 7A, periodontitis was induced by ligating the left maxillary first molar with a 4-0 non-absorbable surgical suture (Ming’an Kang, Yangzhou, China) under intraperitoneal injection of 3% pentobarbital sodium anesthesia at 30 mg/kg. After 21 days, the ligatures were removed and the rats were randomly divided into 4 groups: (1) control group: without any treatment; (2) periodontitis group: ligature-induced periodontitis without treatment; (3) GelMA group: periodontitis rats receiving 20 μL GelMA hydrogel injection; and (4) GelMA@G-Rb3 group: periodontitis rats receiving 20 μL GelMA@G-Rb3 (200 μM) hydrogel injection. Hydrogels were photopolymerized at 405 nm and 25 mW/cm^2^ for 30 s. At 28 days post-treatment, maxillary specimens and major organs, including livers, spleens, and lungs, were collected for analysis.

### 4.10. Micro-CT Analysis

In order to facilitate a more accurate evaluation of the alveolar bone resorption, the molar-maxilla samples were scanned using a Skyscan 1172 micro-CT system (Bruker MicroCT, Kontich, Belgium). Three-dimensional reconstructions were made and analyzed by NRecon 1.6.3 software (Brucker Micro-CT). Additionally, the distance from the enamel-cementum junction (CEJ) to the alveolar crest (AC) between the first molar and the second molar of the rat was determined.

### 4.11. Histological and Immunofluorescence Analysis

Following Micro-CT scanning, maxillary molar specimens underwent standardized processing including 4% paraformaldehyde fixation, EDTA decalcification, graded ethanol dehydration, and paraffin embedding. Mesiodistal sections were prepared at 5-μm thickness, with every fifth section systematically selected to yield three representative sections per sample for histological and immunofluorescence analyses. For histopathological evaluation, sections were stained with hematoxylin and eosin (H&E) to assess general tissue morphology and inflammatory infiltration and Masson’s trichrome to evaluate collagen deposition and fibrosis. For immunofluorescence staining, antigen retrieval was performed using citrate buffer (pH 6.0) at 95 °C for 15 min. Sections were subsequently blocked with rapid blocking solution (Affinity LifeScience, Wuhan, China) for 10 min at room temperature. They were then incubated with primary antibodies against p-p38 (1:150; CST) and p-ERK1/2 (1:150; CST) overnight at 4 °C, followed by Alexa Fluor 488-conjugated secondary antibody (1:1000; Beyotime) for 1 h at room temperature. Nuclei were counterstained with DAPI (1 μg/mL) for 5 min. All stained slides were digitally scanned using Slide Converter 2.3 (3D HISTECH, Budapest, Hungary) and analyzed with CaseViewer software (3D HISTECH).

### 4.12. Statistical Analysis

All data are presented as mean ± SEM with sample sizes ≥3. Statistical analysis was performed using GraphPad Prism 9.5 (GraphPad Software, La Jolla, CA, USA), with one-way ANOVA used to assess differences between groups. A value of *p* < 0.05 was considered statistically significant.

## Figures and Tables

**Table 1:** Gene-specific primer sequences.

Genes	Forward Sequence	Reverse Sequence
IL-6	CAATGAGGAGACTTGCCTGGT	GCAGGAACTGGATCAGGACT
IL-8	CTCTGTGTGAAGGTGCAGTTTT	GTTTTCCTTGGGGTCCAGACA
PTGS 2	ACGCCCTCAGACAGCAAAGC	TGACATGGGTGGGAACAGCAAG
TGF-β	GCAACAATTCCTGGCGATACC	ATTTCCCCTCCACGGCTCAA
GAPDH	CAGGAGGCATTGCTGATGAT	GAAGGCTGGGGCTCATTT

## Data Availability

Data is provided within the manuscript.

## Supplementary Materials

The following supporting information can be downloaded at: https://www.mdpi.com/article/10.3390/gels11080648/s1. Figure S1: The HE staining of liver, spleen, and lung in the animals of different groups.

## Abbreviations

The following abbreviations are used in this manuscript:

AGE-RAGE	advanced glycation end products
ALP	alkaline phosphatase
BP	biological processes
ERK1	extracellular signal-regulated kinase 1
G-Rb3	Ginsenoside Rb3
H&E staining	hematoxylin and eosin staining
IL-6	interleukin-6
LPS	lipopolysaccharide
MA	methacrylic anhydride
MAPK	mitogen-activated protein kinase
Masson staining	Masson’s trichrome staining
Micro-CT	micro computed tomography
MMP2	matrix metalloproteinase 2
PBS	phosphate-buffered saline
PDLSCs	periodontal ligament stem cells
PI3K-Akt	phosphatidylinositol 3-kinase-protein kinase B
PPI	protein–protein interaction
PTGS2	prostaglandin-endoperoxide synthase 2
qPCR	quantitative real-time polymerase chain reaction
SD	Sprague-Dawley
SEM	scanning electron microscopy
SPF	specific pathogen free
TGFβ	transforming growth factor β
