# Supplementary material for: GelMA@ginsenoside Rb3 Targets Inflammatory Microenvironment in Periodontitis via MAPK Pathway
Source: Gels. 2025 Aug 15;11(8):648. doi: 10.3390/gels11080648 (PMC12385488; doi:10.3390/gels11080648)

# GelMA@ginsenoside Rb3 targets inflammatory microenvironment in periodontitis via MAPK pathway

**Jinmeng Sun †, Minmin Sun †, Zekun Li, Luyun Liu, Xinjuan Liu, Yuhui Sun and Gang Ding ***

School of Stomatology, Shandong Second Medical University, Weifang 261053, China; sunjinmeng07@163.com (J.S.); sunminmin@sdsmu.edu.cn (M.S.); zekun_lee@163.com (Z.L.); liuluyun0120@163.com (L.L.); lxj20230834@163.com (X.L.); sunyh03xy@163.com (Y.S.)

* Correspondence: dinggang@sdsmu.edu.cn; Tel.: +86-536-8462450

† These authors contributed equally to this work.

**Supplementary Materials:**

Figure S1: The HE staining of liver, spleen, and lung in the animals of different groups.

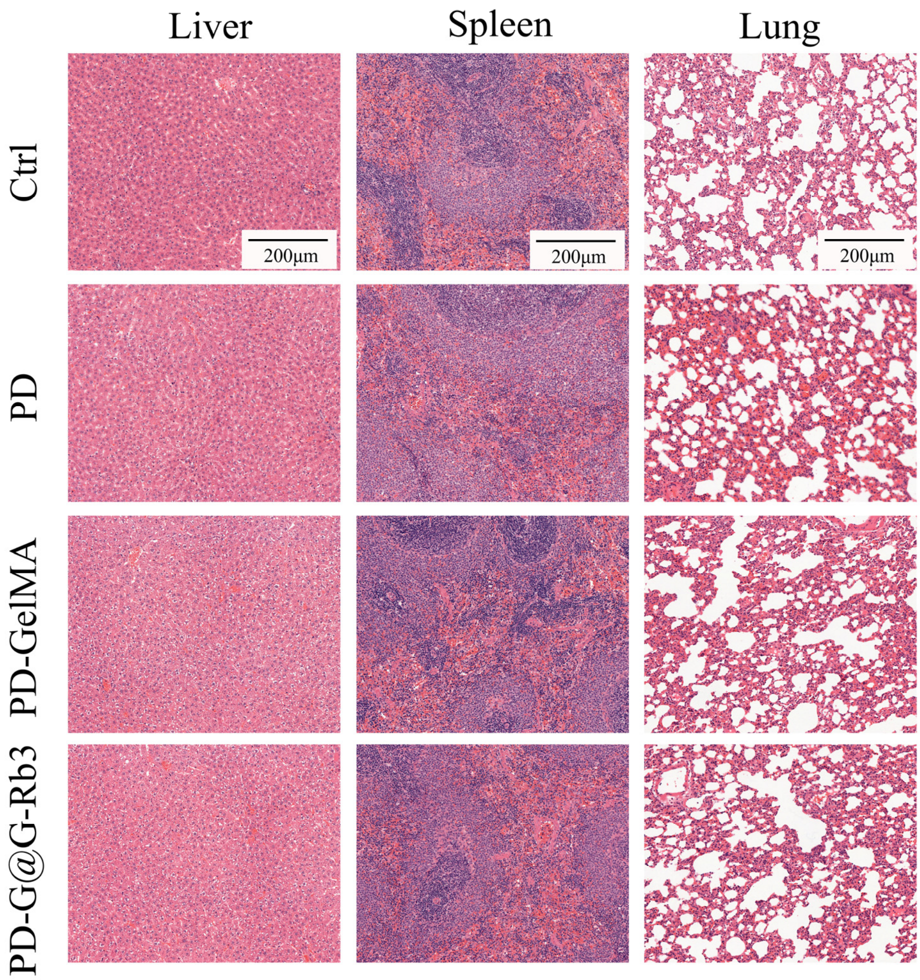

Supplement: Supplementary file 1 [file gels-11-00648-s001.zip › gels-3789482-supplementary.pdf]
